# Risk Factors for DVT/PE in Patients with Stroke and Intracranial Hemorrhage

**DOI:** 10.2174/1874205X01711010060

**Published:** 2017-12-04

**Authors:** Mark Stecker, Kathleen Michel, Karin Antaky, Sarah Cherian, Feliks Koyfman

**Affiliations:** Winthrop University Hospital, Department of Neuroscience, Suite 407, Mineola, NY 11501, USA

The correct names are provided and replaced online which is mentioned as under:

Mark Stecker*, Kathleen Michel, Karin Antaky, Sarah Cherian, Feliks Koyfman

The original names was:

Mark Stecker*, Kathleen Michel, Karin Antaky, Sarah Cherian, Feliks Koyfmann

